# 

**Published:** 1876-10

**Authors:** 


### TO OUR SUBSCRIBERS.

We always feel kindly disposed to our readers—those friends and subscribers who for years have given us their support and encouragement. We have been making a journal for them—one they will find of practical use in times of doubt and difficulty in the daily rounds of practice. We have all these years of our journalism endeavored to be to these toilers a help and a pleasure. We have compiled from all sources the best of practical medical literature for their use at the bedside, that their hands might be strengthened and their hearts cheered in a successful warfare against disease. We have done all this without reward, except the pleasure work of this kind must always bring to those who engage in it. If lives have been saved, if the health of the sick has been restored, then our labor has not been in vain. Now, friends, help us. Help us by writing;—help us by paying your subscriptions;—help us by aiding us in getting subscribers;—help the profession by extending our influence, as well as the sick and afflicted, by the information it gives our brethren. Help us—help one another. Will you not? Go to work; we will do you good.

### BOOK NOTICES.

The Histology and Histochemistry of Man. A treatise on the elments of composition and structure of the human body. By Heinrich Frey, Professor of Medicine in Zurich. Translated from the fourth German edition, by Arthur E. J. Barker, Surgeon to the City of Dublin Hospital, etc., etc. Aud revised by the author, with six hundred and eight illustrations on

### Colaborators and Contributors.

GEORGIA.

T. S. Mitchell, M.D.
J. M. Shaw, M.D.
J. N. Sheny, M.D.
T. M. Tules, M.D.
G. D. Case, M.D.
W. H. Hall, M.D.
S. G. White, M.D.
J. C. Wisdom, M.D.
J. E. Pope, M.D.
E. H. W. Hunter M.D.
Thomas Burdell, M.D.
L. B. Bouchelle, M.D.
W. C. Musgrove, M.D.
W. L. M. Harriss, M.D.
J. M. Tullis, M.D.
G. S. Ullman, M.D.
John Coster, M.D.

KENTUCKY.

Charles Kearns, M.D.
W. S. Taylor, M.D.
Douglass Morton, M.D.

TEXAS.

L. R. Linceeum, M.D.

T. J. Heard, M.D.

TENNESSEE.

F. K. Bailey, M.D.
F. A. Ramsey, M.D.
J. D. Tucker, M.D.

WEST VIRGINIA.

I. . Berry, M.D.
N. D. Tobey, M.D.
P. H. Clark, M.D.
A. L. Knight, M.D.

IOWA.

C. H. Tidd, M.D.
Charles H. Lathrop M.D.

ALABAMA.

J. J. Grace, M.D.
J. D. Rush, M.D.
A. C. Edwards, MD.
S. B. Hogan, M.D.
J. B. Barnett, M.D.
J. C. Knox, M.D.

NORTH CAROLINA.

R. S Hallsey, M.D.
John W. Booth, M.D.
E. A. Anderson, M.D.
George A. Foot M.D.
H. Kelly, M.D.
A. B. Pierce, M.D.
S. S. Satchwell, M.D.
J. B. Hughs, M.D.
Patrick Bootn, M.D.

SOUTH CAROLINA.

G. M. Rivers, M.D.
E. T. McSwain, M.D.

OHIO.

W. P. Wells, M.D.
J. C. Bishop, M.D.
E. H. Trickle, M.D.
W. W. Fierce, M.D.
J. B. Graves, M.D.
J. M. Lucas, M.D.

NEW YORK.

J. S. Baily, M.D.
Ely Van DeWalker, M.D.
Lewis Balch, M.D.
W. H. Vail, M.D.

OREGON.

W. W. Cusick, M.D.

NEW JERSEY.

P. A, Harriss, M.D.
Jno. D. McGill, M.D.
D. M. Forman, M.D.

VIRGINIA.

J. N. Upshur, M.D.
L. B. Anderson, M.D.
W. F. Barr, M.D.
E. A. Drewry, M.D.
S. P. Ripley, M.D
J. S. Wharton, M.D.
R. J. Preston, M.D.
E. Horner, Jr., M.D.

MISSISSIPPI.

E. Cutter, M.D.
T H. Mayo, MD.
I. H. Payne, M.D.
J. M. Lewis, M.D.
A. G Smythe, M.D.
W. T. Beall, M.D.
W. L. Lipscomb, M.D.
Robert Kells, M.D.
E. T. Henry, M.D.
T. P. Lockwood, M.D.
S. V. D. Hill, M.D.
Edward Lee, M.D.

ARKANSAS.

C. D. Hodge, M.D.
A. W. Hobson, M.D.
J. K. Hodge, M.D.
A. D. Hodge, M.D.

INDIANA.

A. Patton, M.D.
G. A. Dyer, M.D.
J. M. Williamson, M.D.

ILLINOIS.

John W. Weir, M.D.

MARYLAND.

A. F. Erich, M.D.

LOUISIANA.

J. W. Wimbish, M.D.

MICHIGAN.

A. E. Hoadley, M.D.

